# Chronic aerobic exercise associated to low-dose L-NAME improves contractility without changing calcium handling in rat cardiomyocytes

**DOI:** 10.1590/1414-431X20198761

**Published:** 2020-03-09

**Authors:** T.C. Luchi, P.M. Coelho, J.P. Cordeiro, A.L.E.M. Assis, B.V. Nogueira, V.B. Marques, L. dos Santos, A.P. Lima-Leopoldo, W. Lunz, A.S. Leopoldo

**Affiliations:** 1 Departamento de Desportos, Centro de Educação Física e Desportos, Universidade Federal do Espírito Santo, Vitória, ES, Brasil; 2 Programa de Pós-Graduação em Nutrição e Saúde, Centro de Ciências da Saúde, Universidade Federal do Espírito Santo, Vitória, ES, Brasil; 3 Departamento de Morfologia, Centro de Ciências da Saúde, Universidade Federal do Espírito Santo, Vitória, ES, Brasil; 4 Departamento de Ciências Fisiológicas, Centro de Ciências da Saúde, Universidade Federal do Espírito Santo, Vitória, ES, Brasil

**Keywords:** Nitric oxide, Aerobic exercise, Cardiac remodeling, Cardiomyocyte, Calcium

## Abstract

Nitric oxide (NO) inhibition by high-dose NG-nitro-L-arginine methyl ester (L-NAME) is associated with several detrimental effects on the cardiovascular system. However, low-dose L-NAME increases NO synthesis, which in turn induces physiological cardiovascular benefits, probably by activating a protective negative feedback mechanism. Aerobic exercise, likewise, improves several cardiovascular functions in healthy hearts, but its effects are not known when chronically associated with low-dose L-NAME. Thus, we tested whether the association between low-dose L-NAME administration and chronic aerobic exercise promotes beneficial effects to the cardiovascular system, evaluating the cardiac remodeling process. Male Wistar rats were randomly assigned to control (C), L-NAME (L), chronic aerobic exercise (Ex), and chronic aerobic exercise associated to L-NAME (ExL). Aerobic training was performed with progressive intensity for 12 weeks; L-NAME (1.5 mg·kg^-1^·day^-1^) was administered by orogastric gavage. Low-dose L-NAME alone did not change systolic blood pressure (SBP), but ExL significantly increased SBP at week 8 with normalization after 12 weeks. Furthermore, ExL promoted the elevation of left ventricle (LV) end-diastolic pressure without the presence of cardiac hypertrophy and fibrosis. Time to 50% shortening and relaxation were reduced in ExL, suggesting a cardiomyocyte contractile improvement. In addition, the time to 50% Ca^2+^ peak was increased without alterations in Ca^2+^ amplitude and time to 50% Ca^2+^ decay. In conclusion, the association of chronic aerobic exercise and low-dose L-NAME prevented cardiac pathological remodeling and induced cardiomyocyte contractile function improvement; however, it did not alter myocyte affinity and sensitivity to intracellular Ca^2+^ handling.

## Introduction

Nitric oxide (NO) is a mediator molecule that acts in multiple functions. It was initially identified as an endothelium-derived relaxing factor and subsequently assigned to many other important biological processes (1). It is synthesized as a free radical from L-arginine by the enzymes nitric oxide synthases (NOS). Three NOS isoforms have been identified, two constitutive and dependent on Ca^2+^ (endothelial (eNOS) and neuronal (nNOS)) and an inducible isoform (iNOS) (1).

NO regulates vascular tonus and influences cardiac function either in physiological or pathological situations (2). NO participates in various events, such as glucose uptake, suppression of collagen synthesis, cell growth and survival, cell contraction, and endogenous inhibition of the maladaptive cardiac hypertrophy signaling cascade (3–5). As a consequence, NO synthesis inhibition leads to hypertension, myocardial hypertrophy with consequent fibrosis and necrosis, as well as cardiomyocyte dysfunction (6,7). In addition, the literature has reported that NOS is targeted to the cardiac sarcoplasmic reticulum and localized in cardiac mitochondria (8). Mitochondrial NOS could be involved in the regulation of cellular functions (8,9).

Several studies have demonstrated that NG-nitro-L-arginine methyl ester (L-NAME) is frequently used to inhibit constitutive NOS in different biological systems (6,7,10,11), reporting that this drug is able to inhibit nearly 100% of all NOS isoforms in the perfused rat heart (10,11). In the cardiovascular system, rats have ingested L-NAME daily, using relatively high (70–250 mg·kg^-1^·day^-1^), moderate (10–50 mg·kg^-1^·day^-1^), and low (1–10 mg·kg^-1^·day^-1^) doses of NOS inhibitors for a period of time varying from 1 to 8 weeks (4,6,7–14). Bernátová et al. (6) showed that chronically administered low-dose L-NAME (1.5 mg·kg^-1^·day^-1^) did not induce deleterious effects to the cardiovascular system of healthy adult rats but did increase NOS activity in aorta and cardiac tissue, suggesting a NO compensatory negative feedback and beneficial effects to the cardiovascular system. Other studies have shown that chronic administration of L-NAME in mice was able to compensate for NOS expression or activity in the myocardium (15,16), possibly by activation of NO via feedback regulatory mechanisms if administered for a longer time (17).

Aerobic exercise is a well-known factor for improving cardiovascular function (18). The hearts of trained individuals exhibit bradycardia, greater myocardial capillarization, and increased contractile force due to improved Ca^2+^ handling of proteins (18,19). Exercise training-induced improvements in vasoreactivity have also been linked with increases in NOS proteins, and could be blocked by L-NAME, supporting this vascular-NO-mediated mechanism of action (20–23); however, the magnitude of NO for beneficial effects induced by exercise has not yet been fully elucidated. Although the model of L-NAME-induced hypertension has been one of the most frequently used experimental models, there is little information on the effects of chronic low-dose L-NAME administration (less than 2 mg·kg^-1^·day^-1^) in healthy hearts subjected to chronic aerobic training. It is possible to speculate two situations from the association between NO inhibition and chronic aerobic exercise: 1) low-dose L-NAME associated with chronic aerobic exercise could promote better myocardium results increasing the isolated beneficial effect of each factor and; 2) this association could lead to effects on healthy hearts that are different from the commonly visualized effects on failing hearts.

The lack of studies and consistent information about the association between low-dose L-NAME and chronic aerobic training in healthy hearts was the impetus for our study. Thus, our purpose was to test whether the association between low-dose L-NAME administration and chronic aerobic exercise promoted beneficial effects to the cardiovascular system through the prevention of pathological cardiac remodeling, hemodynamics, and cardiomyocyte contractility function improvements, as well as positive adjustments in Ca^2+^ handling.

## Material and Methods

### Animals

Adult male Wistar rats (70–100 days of age) obtained from the Animal Quarters of the Federal University of Espírito Santo (Brazil) were housed in collective cages (n=4–5 per cage). The animals were maintained at a constant temperature (22 ± 2°C) on a 12-h reversed light/dark cycle and relative humidity (60±5%) with free access to water and food (Nuvilab CR1-Nuvital, Brazil). Their care and use were carried out in accordance with the Guide for the Care and Use of Laboratory Animals published by the U.S. National Institutes of Health, as well as with current Brazilian laws. All procedures were approved by the Institutional Ethics Committee on Animal Use (CEUA-UFES 050/2012).

### Experimental Design

Rats were randomly assigned to four experimental groups: chronic aerobic exercise (Ex; n=14), submitted to continuous aerobic exercise on treadmills; L-NAME (L; n=14), submitted to low-dose administration of L-NAME; chronic aerobic exercise + L-NAME (ExL; n=16), concomitantly submitted to continuous aerobic exercise and administration of low dose L-NAME; and control (C; n=12), submitted to the same treatment as the other groups with the exception of exercise and administration of L-NAME interventions. The duration of the experimental protocol was 12 weeks.

### L-NAME administration

N^G^-nitro-L-arginine methyl ester (L-NAME, purchased from Sigma Chemical Co., USA) is a non-specific NO synthase inhibitor, commonly used for the induction of NO-deficient hypertension (6). The inhibitory effect of L-NAME is dose-dependent and time-dependent with a short L-NAME half-life (min), but its effects are prolonged if hydrolyzed in Nω-nitro-l-arginine (L-NNA) (14). L-NAME was administered daily in the morning (coinciding with the training days) by orogastric gavage for 12 weeks at a dose of 1.5 mg·kg^-1^·day^-1^. Body weight was measured every other day to estimate L-NAME dose. At the 12th week of the experimental protocol, administration of L-NAME was terminated, preceding the final performance test. The inclusion of L-NAME in the drinking water did not alter water intake. In the C and Ex groups, the animals received water by gavage (approximately 0.2 mL) throughout the entire experiment to be submitted to the same stress.

### Maximum running speed (MRS) test

Before starting the aerobic training protocol, the animals were placed on the treadmill for acclimatization (10 min/day, 0% grade, 10 m/min) for 7 days. After adaptation, all rats were submitted to the treadmill exercise test (15 m/min, 10 min, 0% grade, for 5 days). Those animals that did not complete this preliminary test were randomly selected for C (n=12) or L (n=14) groups. The animals that completed the preliminary test were randomly selected for Ex (n=14) or ExL (n=16) and submitted to the MRS test to adjust the load at weeks 4, 8, and 12. The MRS test was carried out at 10 m/min, 0% grade, and the running speed was increased by 3 m/min every 3 min (10 to 34 m/min.). The test was performed until the rat was unable to maintain the pace of the treadmill belt. The highest speed which the rat could maintain for 15 s was defined as MRS (18). At the end of 12 weeks, all of the animals were submitted to a final MRS test. C and L animals remained cage-confined without any physical training program throughout the 12-week protocol. The following parameters were evaluated: speed (m/min), duration (min), and distance (m). Exercise motivation was provided by a mild electronic shock grid (0.2 mA) at the rear of the treadmill during the MRS test and aerobic training protocol.

### Aerobic training protocol

The training intensity was determined by the MRS test. The animals of the Ex and ExL groups were submitted to the progressive aerobic training protocol in a motor-driven treadmill (Insight Instruments, Brazil). From weeks 1 to 5, aerobic training was performed 5 days/week with a progressive increase of duration (15 to 60 min/day) and intensity (50 to 60%). From the 6th week, physical exercise was performed 3 days/week due to the high mortality rate of the ExL group (approximately 90%); this result was also verified in a previous study using a high-dose L-NAME (4). To maintain training volume in the 6th week, exercise duration was augmented in 60 min/day and intensity was kept constant (60%). In the seventh, eighth, and ninth weeks, the rats trained 3 days/week with a progressive increase of duration (15 to 60 min/day) and 70% of intensity. Finally, in the three last weeks, the trained rats performed the aerobic exercise training with the same frequency and duration of previous weeks (7 to 9), but the intensity was 80%. During the training period, animals from the C and L group were placed on the treadmill 5 days a week at similar times as Ex and ExL without performing exercise. All procedures were performed during the morning (9:30–11:30 am).

### Adiposity parameters

After 12 weeks of the experimental protocol, rats were anesthetized intraperitoneally with ketamine hydrochloride (50 mg/kg *ip*, Dopalen, Sespo Indústria e Comércio Ltda., Vetbrands Division, Brazil) and xylazine hydrochloride (10 mg/kg *ip*, Anasedan, Sespo Indústria and Comércio Ltda., Vetbrands Division). Following this, their chests were opened by mid-thoracotomy and the adipose tissue fat pads were dissected and weighed. The adiposity index, used to assess adiposity, was calculated using the following formula: adiposity index (body fat (BF)/final body weight) ×100. BF was measured from the sum of the individual fat pad weights as follows: BF = epididymal fat + retroperitoneal fat + visceral fat.

### Blood pressure measurements

Systolic and diastolic blood pressure (SBP and DBP), mean arterial pressure (MAP), and heart rate (HR) were measured in conscious rats using an indirect non-invasive tail-cuff method (IITC INC, Life Science, USA) at 4, 8, and 12 weeks of experiment. Three stable consecutive readings were taken and recorded for each animal.

### Hemodynamics measurements

At the end of the 12-week training protocol, rats were anesthetized intraperitoneally with ketamine hydrochloride (50 mg/kg *ip*) and xylazine hydrochloride (10 mg/kg *ip*) (Dopalen, Sespo Indústria e Comércio Ltda., Vetbrands Division) and submitted to catheterization surgery. Hemodynamic data were obtained by a micromanometer (MikroTip^TM^ SPR 320, Millar Instruments, USA) inserted from the right carotid artery and positioned immediately above the aortic valves to monitor the aortic and LV pressure records. SBP and DBP (mmHg), HR (bpm), LV systolic and end-diastolic pressures (LVSP and LVEDP; mmHg), maximum positive (+dP/dtmax; mmHg/s) and negative (-dP/dtmax; mmHg/s) derivatives of LV pressures, and LV relaxation time constant (TAU; s) were acquired and analyzed using a computer program (Acknowledge Software, Biopac System, USA).

### Euthanasia and tissue samples

At the end of the experimental protocol, the animals received an injection of sodium heparin (1000 U/kg *ip*; Heparamax-s, Blau Pharmaceutic S.A., Brazil) for cardiomyocyte analysis. After 30 min, rats were anesthetized with ketamine plus xylazine (50+10 mg/kg, *ip*) and euthanized by decapitation. Subsequently, their chests were opened by mid-thoracotomy, and the heart, ventricles, atrium, fat pads, and tibia were separated, dissected, and weighed or measured.

### Morphological analysis

Cardiac remodeling at the macroscopic level, which identifies the presence or absence of cardiac hypertrophy, was determined by analyzing the following parameters: heart weight (HW), LV, right ventricle (RV), and atrium (AT) weights, and HW/, LV/, and RV/tibia length ratios.

### Histology

LV transverse sectional areas of animals from each group were fixed in phosphate-buffered 4% paraformaldehyde (pH 7.4) and embedded in paraffin. Sections of 6 µm were obtained and stained with hematoxylin-eosin (HE) and picrosirius red stain to determine the myocyte cross-sectional area and to measure the collagen volume fraction, respectively. Images were captured with a video camera coupled to an optical microscope (AX70, Olympus Optical Co., Germany) under 40× or 20× objective, which sends digital images to a computer with an image analysis program (Image Pro-plus, Media Cybernetics, USA). A total of 30–50 myocytes per animal were used to determine the mean cross-sectional area (CSA; µm^2^). The myocytes were chosen in transverse section, with a clearly visible nucleus occupying the central region of the cell.

The collagen area fraction (%) was determined using 15 microscopic fields per fragment. The components of the cardiac tissue were identified according to color level as follows: red for collagen fibers, yellow for myocytes, and white for interstitial space.

### Cardiomyocyte preparation

Under anesthesia, rats from each group were killed and their hearts quickly removed by median thoracotomy and enzymatically isolated as previously described (24). Briefly, the hearts were cannulated via the aorta and perfused in the Langendorff system (37°C) with a modified digestion buffer solution (DB), a calcium-free solution with EGTA (0.1 mM), and HEPES equilibrated with 5% CO_2_-95% O_2_ for ∼3 to 5 min. The composition of the DB solution was (mM): 130 NaCl, 1.4 MgCl_2_, 5.4 KCl, 25 HEPES, 22 glucose, 0.33 NAH_2_PO4, and pH 7.39. Afterward, the hearts were perfused for 15–20 min with a DB solution containing 1 mg/mL collagenase type II (Worthington Biochemical Corporation, UK) and Ca^2+^ (1 mM). The digested hearts were then removed from the cannula, cut down, and placed into small conical flasks with the DB solution containing collagenase supplemented with 0.1% bovine serum albumin and Ca^2+^ (1 mM). This process was performed twice without collagenase, and at each stage the tube containing the cells and solutions was held for approximately 10 min and the supernatant discarded. The resulting cell was resuspended in Tyrode solution of the following composition (mM): 140 NaCl, 10 HEPES, 0.33 NaH_2_PO_4,_ 1 MgCl_2_, 5 KCl, 1.8 CaCl_2_, and 10 glucose. Non-dispersed tissue was subjected to further enzyme treatment. Only calcium-tolerant, quiescent, rod-shaped cardiomyocytes showing clear cross-striations were studied. The isolated cardiomyocytes were used within 2-3 h of isolation.

### Cardiomyocyte contractility

Isolated cells were placed in an experimental chamber with a glass coverslip that was base-mounted on the stage of an inverted microscope (IonOptix, USA) edge detection system with a 40× objective lens (Nikon Eclipse – TS100, USA). Cells were immersed in Tyrode's solution containing 1.8 mM CaCl_2_ and the field was stimulated at 1 Hz (20 V, 5-ms duration square pulses). Cell shortening in response to electrical stimulation was measured with a video-edge detection system at a 240-Hz frame rate (IonWizard, IonOptix, USA) and the contractile parameters were evaluated. Fractional shortening (expressed as a percentage of resting cell length) and times to 50% shortening and relaxation were measured in 15–20 cells per animal in each experimental group.

### Intracellular Ca^2+^ measurements

Myocytes were loaded with 1.0 μM Fura 2-acetoxymethyl (AM) ester (Molecular Probes, USA) for 10 min at room temperature, washed with Tyrode solution, and allowed to rest for an additional 10 min to allow the desesterification of dye. Subsequently, the cardiomyocytes were stimulated at 1 Hz (Myopacer 100, IonOptix) and fluorescence images were obtained using excitation of 340 to 380 nm wavelengths using a Hyper Switch system (IonOptix). Background-corrected Fura 2AM ratios, reflecting intracellular Ca^2+^ concentration, were detected at approximately 510 nm. The Ca^2+^ transient amplitude was reported as F/F0, where F is the maximal fluorescence intensity average measured at the peak of [Ca^2+^]i transients and F0 is the baseline fluorescence intensity measured at the diastolic phase of [Ca^2+^]i transients. Time to 50% peak of transient Ca^2+^ and time to 50% Ca^2+^ decay were also analyzed.

### Statistical analysis

Data on general characteristics, cardiac remodeling, and cardiomyocytes are reported as mean ± standard error of mean (SEM) and were submitted to the Kolmogorov-Smirnov test to determine adherence to normality. Comparisons of the maximum velocity tests were made by Student's *t*-test for independent samples. A two-way analysis of variance (ANOVA) was utilized to evaluate the effects of chronic aerobic exercise and low-dose administration of L-NAME on cardiac remodeling, contractile parameters, and Ca^2+^ transient in isolated cardiomyocytes (C, L, Ex, and ExL). When significant differences were found (P<0.05), a Tukey *post hoc* was carried out. The level of significance was 5%.

## Results

**Figure 1.**
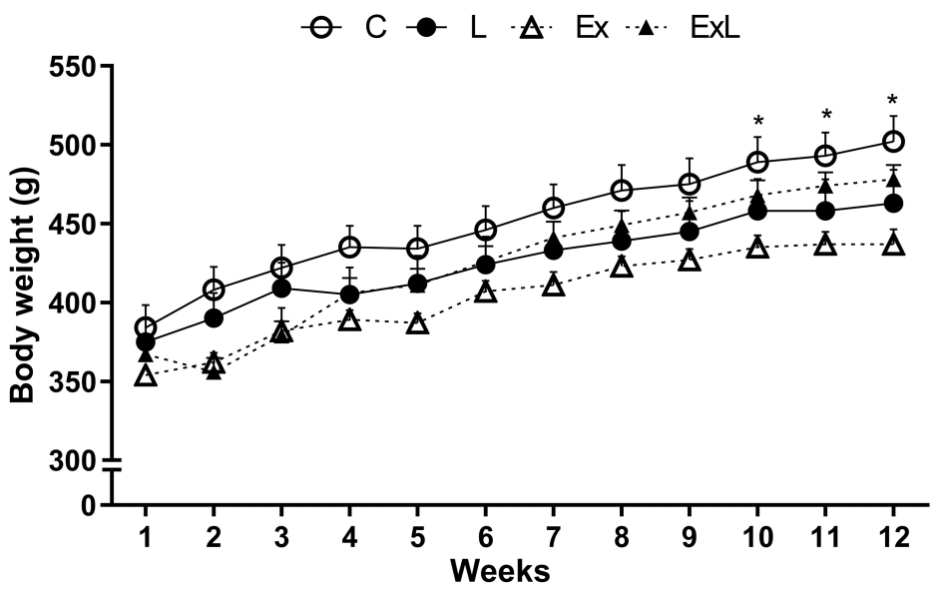
Evolution of body weight during 12 weeks of experiment. Data are reported as mean±SEM. C: Control (n=12); L: low-dose administration of L-NAME (n=14); Ex: chronic aerobic exercise (n=14); ExL: chronic aerobic exercise and low-dose administration of L-NAME (n=16). *P<0.05, C *vs* Ex (two-way ANOVA for repeated measures followed by Tukey's *post hoc* test).

Chronic aerobic exercise (Ex) promoted a substantial reduction of final BW (11 to 12%) in relation to C from the 10th week of treatment (P<0.05), with this difference remaining until the 12th week (Figure 1). There was no statistical difference for BW among the other groups over the 12 weeks of the experimental protocol. In addition, the initial BW was similar among the groups.

Chronic aerobic exercise (Ex) led to a significant reduction in final BW, weight gain, visceral, retroperitoneal and epididymal fat pads, total BF, and adiposity index (AI) in relation to group C. Similarly, the administration of low doses of L-NAME caused lower BW gain, retroperitoneal and epididymal fat pads, total BF, and AI than in group C, but there were no alterations in the final BW (L= 463±21 *vs* C: 502±16; P>0.05) and visceral fat pad (L: 5.97±0.69 *vs* 7.21±0.65, P>0.05) between these groups. Specifically, the ExL rats had a significantly higher final BW (ExL: 478±9 *vs* Ex: 437±9), weight gain (ExL: 111±7 *vs* Ex: 83±8), retroperitoneal fat pads (ExL 8.37±0.55 *vs* Ex: 5.59±0.45), BF (ExL: 18.4±1.0 *vs* Ex: 12.9±1.0), and AI (ExL: 3.85±0.20 *vs* Ex: 2.94±0.22) compared to Ex rats, indicating an elevation of 9.4, 33.7, 49.7, 42.6, and 31%, respectively. In addition, the ExL group showed no differences in relation to the L group for all variables (Table 1).

**Table 1.** General characteristics.

Variables	Experimental Groups			
	C (n=12)	L (n=14)	Ex (n=14)	ExL (n=16)
Initial body weight (g)	384±14	375±15	354±6	367±9
Final body weight (g)	502±16	463±21	437±9*	478±9^#^
Weight Gain (g)	118±10	88±8*	83±8*	111±7^#^
Visceral fat pad (g)	7.21±0.65	5.97±0.69	4.46±0.43*	5.87±0.34
Retroperitoneal fat pad (g)	13.5±1.5	9.93±1.23*	5.59±0.45*	8.37±0.55^#^
Epididymal fat pad (g)	6.04±1.02	4.05±0.63*	2.82±0.34*	4.13±0.23
Body fat (g)	26.7±2.7	20.0±2.4*	12.9±1.0*	18.4±1.0^#^
Adiposity Index (%)	5.25±0.41	4.16±0.34*	2.94±0.22*	3.85±0.20^#^

Data are reported as mean±SEM. C: Control; L: low-dose administration of L-NAME; Ex: chronic aerobic exercise; ExL: chronic aerobic exercise and low-dose administration of L-NAME; n: number of animals. *P<0.05 *vs* C; ^#^P<0.05 *vs* Ex (two-way ANOVA followed by Tukey's *post hoc* test).

**Figure 2.**
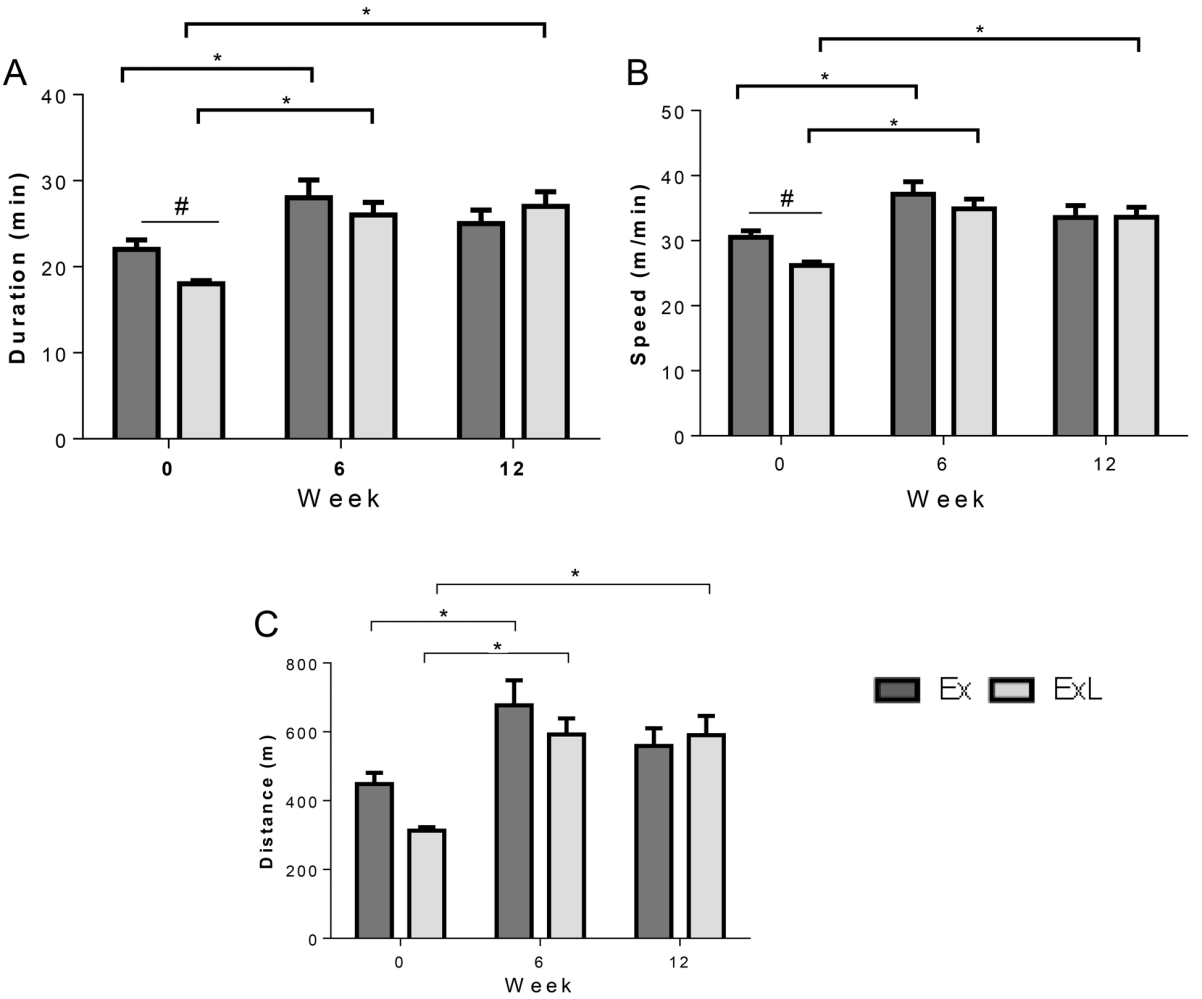
Maximum running speed test performed in exercised groups. Ex: chronic aerobic exercise (n=14) and ExL: chronic aerobic exercise and low-dose administration of L-NAME (n=16). Data are reported as mean ± SEM. *P<0.05, *vs* 0 week; ^#^P<0.05, Ex *vs* ExL (one-way ANOVA followed by Tukey's *post hoc* test).

Groups submitted to chronic aerobic exercise (Ex and ExL) displayed an increase in duration, speed, and distance during the MRS test at week 6 compared to the week 0 baseline (Figure 2). In relation to the ExL group, there were also differences at week 12 *vs* week 0, since this group presented increased duration, distance, and speed. There were alterations in duration (Ex: 22±1 min *vs* ExL: 18±0.4 min; P<0.015) and speed (Ex: 31±1 m/min *vs* ExL: 26±0.5 m/min; P<0.021) at week 0 between Ex and ExL, respectively (Figure 2A and B). In addition, the results showed there was no significant difference in completed distance between Ex groups (Figure 2C).

**Figure 3.**
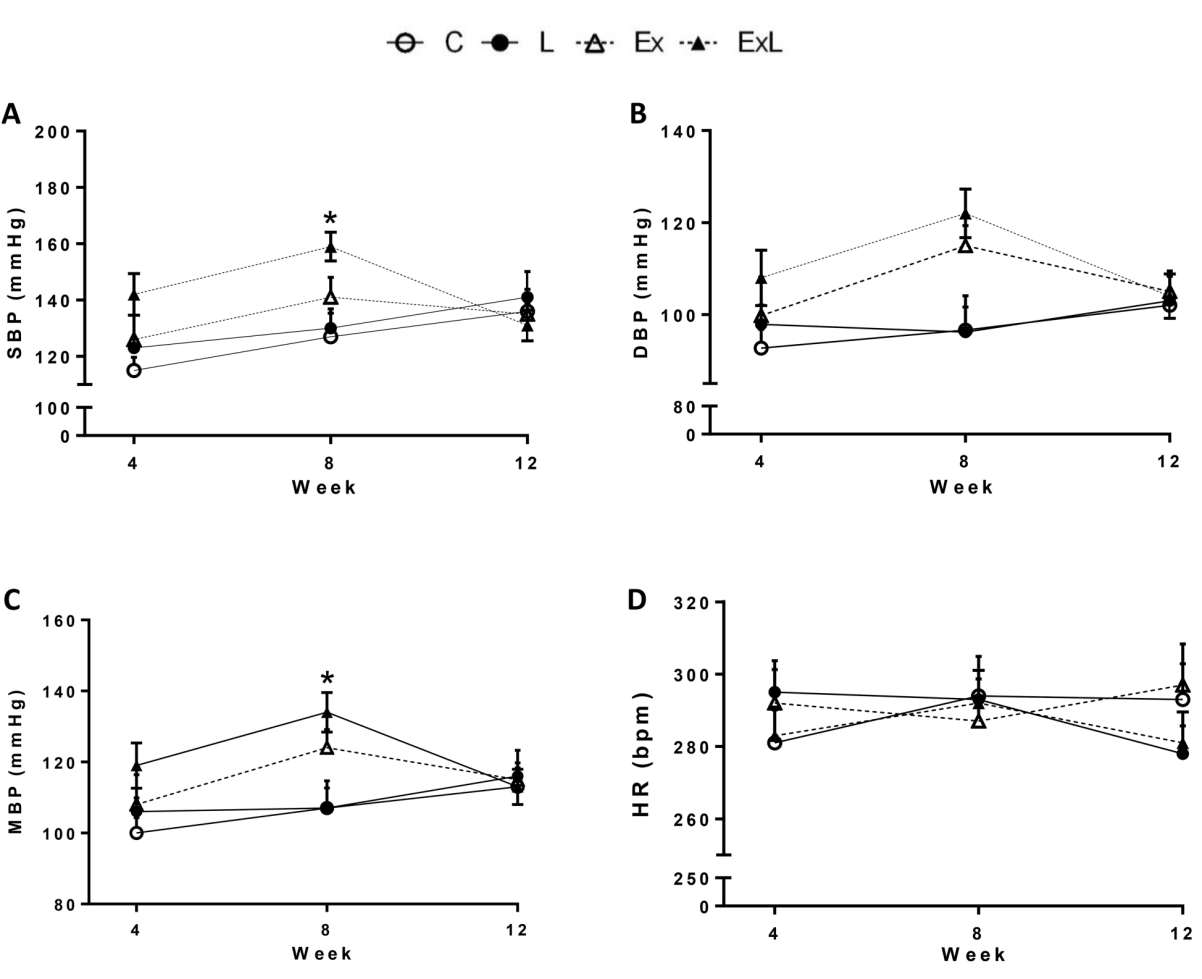
Blood pressure responses and heart rate during 12 weeks of experimental protocol. C: Control (n=8); L: low-dose administration of L-NAME (n=7); Ex: chronic aerobic exercise (n=6); ExL: chronic aerobic exercise and low-dose administration of L-NAME (n= 6). **A**, SBP: systolic blood pressure; **B**, DBP: diastolic blood pressure; **C**, MAP: mean arterial pressure; **D**, HR: heart rate. Data are reported as mean±SEM. *P<0.05, ExL *vs* L (two-way ANOVA followed Tukey's *post-hoc* test).

Administration of low-dose L-NAME associated with chronic aerobic exercise (ExL) presented a significantly increased SBP (ExL: 159±6 mmHg *vs* L: 130±7 mmHg; P<0.049) and MBP in relation to L (ExL: 134±6 mmHg *vs* L: 107±6 mmHg; P<0.02) at week 8 (Figure 3A and C), but these parameters were normalized at week 12. In addition, DBP and HR did not show significant differences among the groups.

The association of chronic aerobic exercise and low-dose L-NAME promoted significantly enhanced LVEDP compared to L and Ex (Table 2). Specifically, the ExL rats presented an elevation of 50 and 38% in LVEDP in relation to L and Ex, respectively. This hemodynamic change was not accompanied by significant differences in HR, TAU, LVSP, +dP/dT_máx_, and -dP/dT_máx_ (ExL *vs* L and Ex; P>0.05). In addition, these variables were not significantly different between C and the L and Ex groups (Table 2).

**Table 2.** Hemodynamics measurements.

Variables	Experimental Groups			
	C (n=5)	L (n=6)	Ex (n=7)	ExL (n=6)
LVSP (mmHg)	127±9	126±13	143±4	143±8
LVEDP (mmHg)	5.74±1.23	7.20±2.14	9.22±1.48	15.0±1.70^#&^
+dP/dt_max_ (mmHg/s)	6843±218	6268±431	6918±385	6976±541
-dP/dt_max_ (mmHg/s)	-5870±109	-5860±348	-6464±207	-5929±405
Tau (s)	0.02±0.002	0.02±0.002	0.03±0.004	0.02±0.001
Heart rate (bpm)	227±10	227±7	207±5	212±5

Data are reported as mean±SEM. C: control; L: low-dose administration of L-NAME; Ex: chronic aerobic exercise; ExL: chronic aerobic exercise and low-dose administration of L-NAME; n: number of animals; LVSP: left ventricle systolic pressure; LVEDP: LV end-diastolic pressure; +dP/dt_max_: maximum positive derivative of LV pressure; -dP/dt_max_: maximum negative derivative of LV pressure; Tau: LV relaxation time constant. ^#^P<0.05 ExL *vs* Ex; ^&^ExL *vs* L (two-way ANOVA followed by Tukey's *post hoc* test).

**Figure 4.**
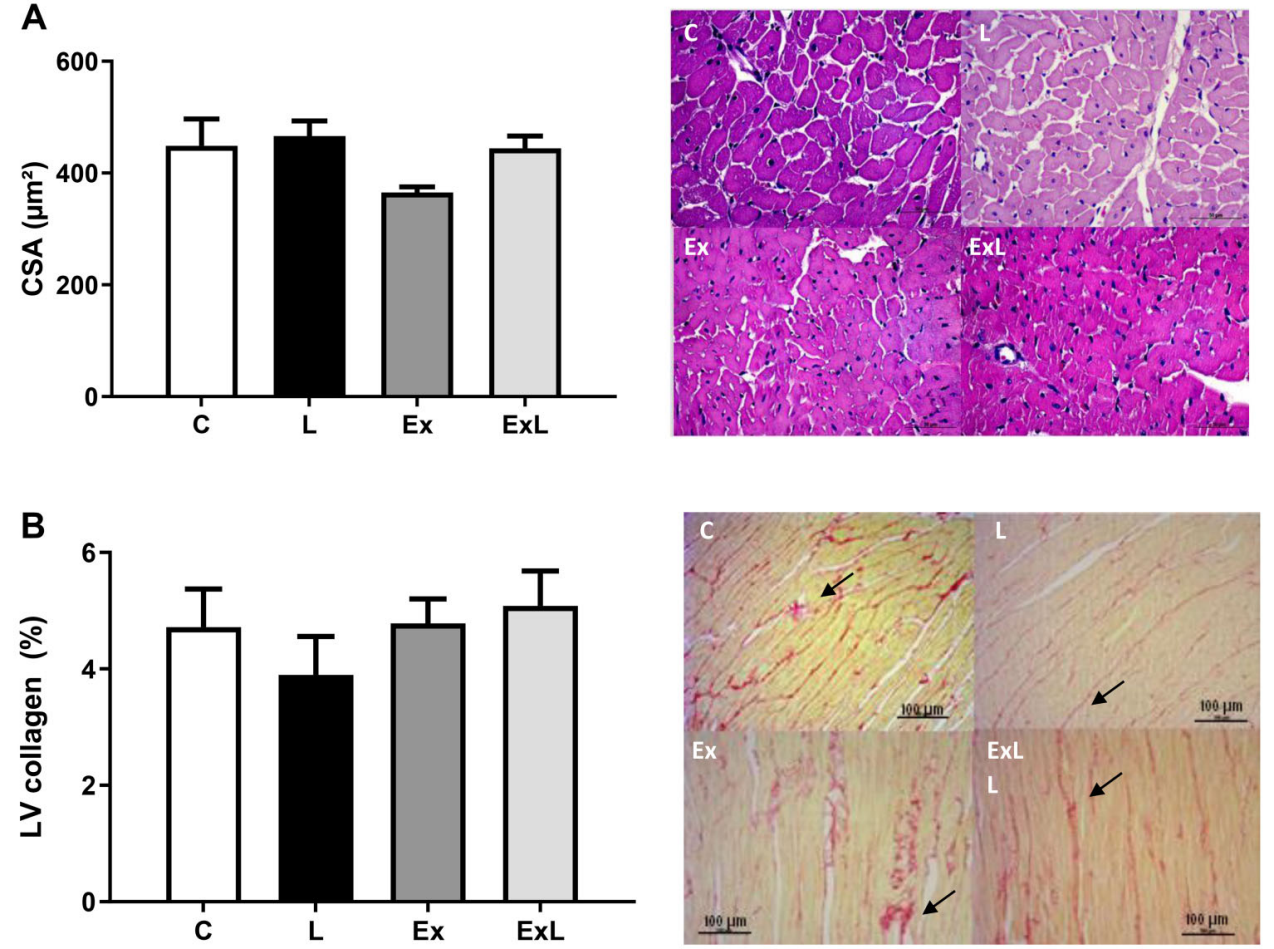
Histological studies performed in C: Control (n=6); L: low-dose administration of L-NAME (n=7); Ex: chronic aerobic exercise (n=5); ExL: chronic aerobic exercise and low-dose administration of L-NAME (n=10). LV: **A**, CSA: cross-sectional area obtained by HE staining for reticulin (magnification 40×, magnification bar 50 μm). **B**, Interstitial collagen of myocardium in representative picrosirius red-stained left ventricle (LV) section (magnification 20×, magnification bar 100 μm). Arrows: interstitial collagen. Data are reported as mean±SEM. P>0.05, two-way ANOVA followed by Tukey's *post-hoc* test.

Absolute AT weight and AT-to-tibia length ratio were significantly elevated in ExL rats compared with those in the Ex group. This association also promoted elevation of HW-to-tibia length ratio compared to L rats (Table 3). The Ex rats also presented lower AT and AT-to-tibia length than the C group. Additionally, the histological analysis did not reveal differences in CSA and LV interstitial collagen among the groups (Figure 4A and B).

**Table 3.** Morphological characteristics.

Variables	Experimental Groups			
	C (n=6)	L (n=7)	Ex (n=5)	ExL (n=10)
Heart weight (g)	1.26±0.04	1.18±0.06	1.18±0.07	1.27±0.05
Left ventricle weight (g)	0.89±0.02	0.85±0.05	0.87±0.05	0.90±0.03
Right ventricle weight (g)	0.25±0.03	0.21±0.02	0.22±0.01	0.24±0.01
Total atrium weight (g)	0.13±0.01	0.12±0.01	0.09±0.01*	0.13±0.01^#^
Tibia length (cm)	4.10±0.04	4.19±0.07	4.10±0.03	3.98±0.05
HW/tibia length (g/cm)	0.31±0.01	0.28±0.01	0.29±0.02	0.32±0.01^&^
LV/tibia length (g/cm)	0.22±0.01	0.20±0.01	0.21±0.01	0.23±0.01
RV/tibia length (g/cm)	0.061±0.007	0.051±0.004	0.054±0.004	0.060±0.003
AT/tibia length (g/cm)	0.031±0.002	0.029±0.002	0.022±0.002*	0.032±0.002^#^

Data are reported as mean±SEM. C: Control; L: low-dose administration of L-NAME; Ex: chronic aerobic exercise; ExL: chronic aerobic exercise and low-dose administration of L-NAME; n: number of animals; HW: heart weight; LV: left ventricle weight; RV: right ventricle weight; AT: total atrium weight; *P<0.05 *vs* C; ^#^P<0.05 ExL *vs* Ex; ^&^P<0.05 ExL *vs* L (two-way ANOVA followed by Tukey's *post hoc* test).

**Figure 5.**
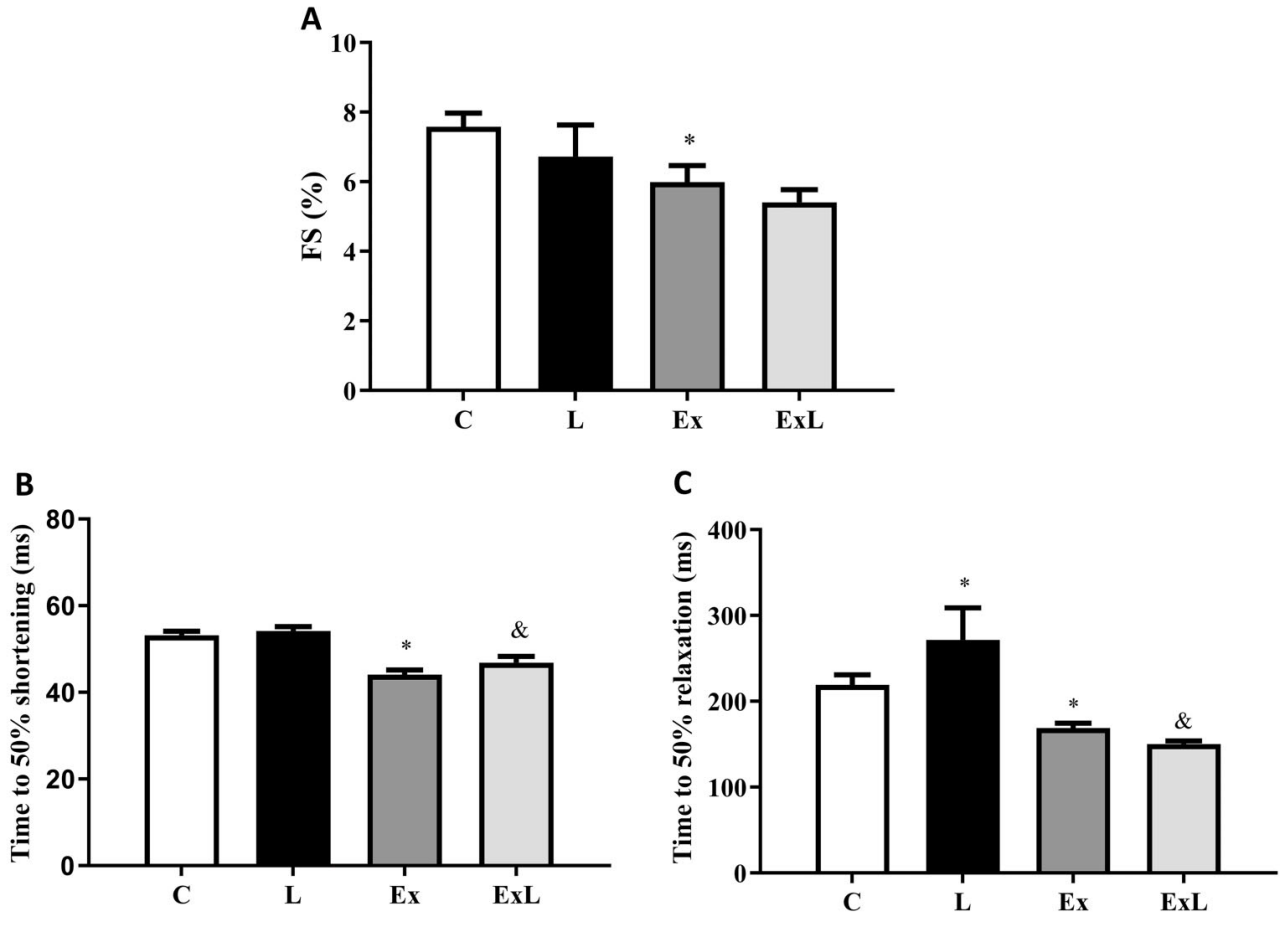
Contractile responses of cardiomyocytes from C: control (n=5; 42 cells); L: low-dose administration of L-NAME (n=4; 51 cells); Ex = chronic aerobic exercise (n=6; 111 cells); ExL: chronic aerobic exercise and low-dose administration of L-NAME (n=5; 94 cells). **A**, FS (%): fractional shortening: **B**, time to 50% shortening and **C**, relaxation. Data were obtained from electrical stimulation with 1 Hz and are reported as mean±SEM. *P<0.05 *vs* C; ^&^P<0.05 ExL *vs* L (two-way ANOVA followed by Tukey's *post hoc* test).

**Figure 6.**
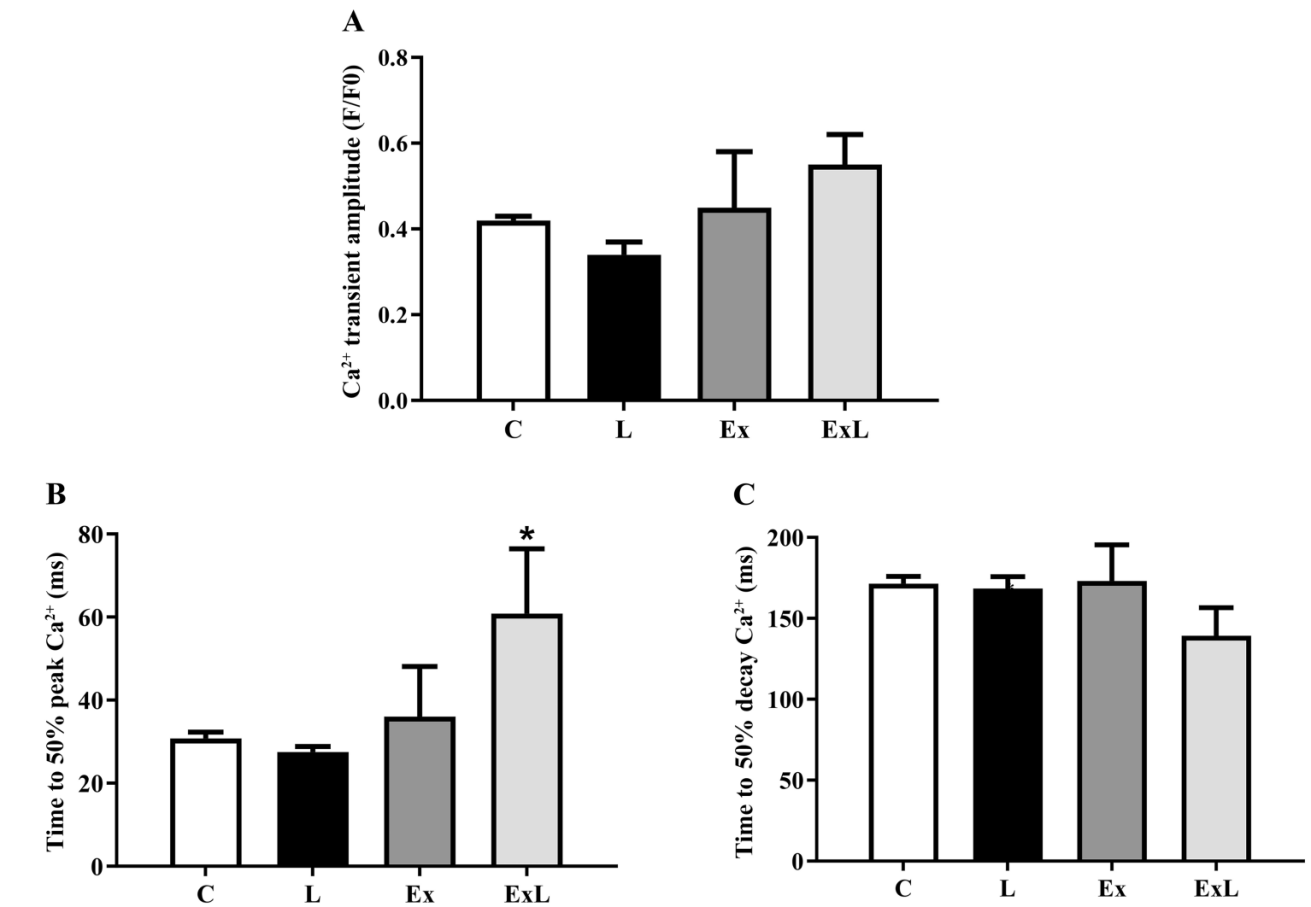
Cardiomyocyte calcium transients. Data are reported as mean±SEM. C: control (n=4; 25 cells); L: low-dose administration of L-NAME (n=4; 17 cells); Ex: chronic aerobic exercise (n=4; 14 cells); ExL: chronic aerobic exercise and low-dose administration of L-NAME (n=5; 7 cells). **A**, Ca^2+^ transient amplitude. **B**, time to 50% peak Ca^2+^, and **C**, time to 50% decay Ca^2+^. The records were obtained from electrical stimulation with 1 Hz. *P<0.05 ExL *vs* L (two-way ANOVA followed by Tukey's *post hoc* test).

Figures 5 and 6 summarize the mechanical properties of isolated cardiomyocytes and intracellular Ca^2+^ transients from all groups. Low-dose L-NAME when associated with chronic aerobic exercise induced a marked adaptive response in cardiomyocyte contractile function and Ca^2+^ handling. Fractional shortening was reduced by 24.4% in the ExL group in relation to L, but no difference was observed (P>0.05). Nevertheless, cardiomyocytes from ExL animals exhibited shorter times to 50% shortening and relaxation (16 and 81%, respectively) compared with those from L. In addition, L rats exhibited a significantly longer time to 50% relaxation than C rats (Figure 5C). Moreover, Ex group had reduced fractional shortening as well as times to 50% shortening and relaxation in relation to group C (Figure 5A–C). The results from Ca^2+^ handling showed that the time to 50% Ca^2+^ peak was increased in ExL compared to L (Figure 6B). No differences were observed in Ca^2+^ transient amplitude and time to 50% Ca^2+^ decay among groups (Figure 6A and C).

## Discussion

Although the administration of low-dose L-NAME seems to offer benefits to the cardiovascular system (6), the effects of high doses of L-NAME have been well studied as an experimental model of hypertension, and its deleterious effects are demonstrated in the literature (7,12,13,25,26). Despite this, beneficial effects of low-dose L-NAME were reported in normotensive healthy rats without any other intervention. Aerobic exercise is a well-known factor for improving cardiovascular function, visualized by NO synthase proteins increased with consequent elevation of NO production, supporting this vascular NO-mediated mechanism of action. The hypothesis tested in the current study was that the association of L-NAME administered in low doses to healthy animals submitted to chronic aerobic training would promote beneficial effects to the cardiovascular system, with prevention of pathological cardiac remodeling, hemodynamics, and cardiomyocyte contractility function improvements, as well as beneficial effects on Ca^2+^ handling. The main finding of our study was that the association of exercise with low-dose L-NAME promoted damage to the heart, including elevated LVEDP and myocardial fibrosis associated with impaired cardiomyocyte contractility and Ca^2+^ handling. To our knowledge, this was the first study to evaluate the effects of aerobic exercise combined with the administration of low-dose L-NAME on cardiac remodeling and function, as well as on contractile and Ca^2+^ handling parameters of isolated cardiomyocytes.

Regular physical exercise promotes several benefits, influencing metabolism and caloric expenditure and modifying body composition. Our results show that 12 weeks of aerobic training decreased weight gain and total body fat (30 and 51%, respectively). The association of aerobic exercise with low doses of L-NAME was not able to prevent alterations in body composition compared to L and C. Sansbury and Hill (27) have reported that the gaseous signaling molecule NO may play a pivotal role in regulating systemic metabolism and body composition, demonstrating that NO bioavailability could be decreased via reduced expression of NOS enzymes. In rodents, NOS inhibitors decreased food intake (28,29) due to NO activity in the brain, impinging on the leptin and serotonergic systems that regulate hunger. According to literature, NOS inhibitors, such as L-NAME, promote weight loss and diminish food intake in obese rats (29,30), as well as reduce adiposity after consumption of a high-fat diet. Therefore, it would seem that NO produced in the brain antagonizes anorectic signals and stimulates food intake with consequent weight gain (27–30), while L-NAME promotes an orexigenic effect and a reduction of adiposity parameters as observed in our study. Possible explanations for non-prevention of adiposity when associated with chronic aerobic exercise (ExL) are the short time of the intervention (12 weeks) and the lower energy expenditure during caged rest time. We did not evaluate the motor behavior of animals beyond exercise time.

The administration of L-NAME is widely used as a model of experimental hypertension (31). It is interesting to note that the magnitude of the increase in BP and the deleterious effects generated by the blockade of NO synthesis depends on the way L-NAME is administered, the dose, as well as the treatment period (7,17,32), as treatment with high-dose L-NAME is often associated with heart abnormalities such as arterial hypertension (7,32). In the present study, neither low-dose L-NAME nor chronic aerobic exercise associated with low-dose L-NAME were able to significantly change blood pressure at the end of the experiment. Nevertheless, our data indicated that the association between low doses of L-NAME and chronic aerobic exercise leads to an increase in SBP at the 8th week of protocol, although this alteration was normalized after 12 weeks, suggesting that this association did not promote arterial hypertension after long-term administration of L-NAME even with LVEDP increased at the 12th week in relation to Ex and L.

In relation to the isolated effect of NO inhibition on aerobic exercise capacity at baseline and over time, few studies have been conducted and generally show that the acute inhibition of NOS by L-NAME is known to decrease maximal oxygen consumption (VO_2_max) and impair maximal exercise capacity, whereas the effects of chronic L-NAME treatment on VO_2_max and exercise performance have not been studied so far, as well as L-NAME associated to exercise (33). Wojewoda et al. (33) showed that short-term (2 weeks) but not long-term (12 weeks) treatment with L-NAME activates robust compensatory mechanisms involving preservation of plasma nitrite (NO_2_) concentration, overproduction of prostacyclin (PGI2), and increased number of circulating erythrocytes (RBC), which might explain the fully preserved exercise capacity despite the inhibition of NOS. Our results are in accordance to Wojewoda et al. (33), however the biomarkers were not evaluated in the current study.

The literature reports two types of cardiac remodeling: physiological or well-adapted, in which morphological and functional adaptations occur without damage to the cardiac function; and pathological or maladaptive, associated with an increase in LVEDP, pulmonary congestion, and LV hypertrophy (34). Studies have shown the influence of the anti-hypertrophic role of NO (5,32). In this sense, the association of chronic aerobic exercise with the administration of L-NAME promoted increased atrium weights, suggesting atrial remodeling. Our data are in disagreement with Goessler et al. (35) who found cardiac remodeling in Wistar rats treated with 20 mg/kg of L-NAME administration associated with aerobic exercise after 4 weeks. In addition, high-dose L-NAME increased cardiomyocyte number and size, as well as LV mass (32). Other studies have demonstrated an increase in cardiac macroscopic areas after 7 days of high-dose L-NAME administration (70 mg/kg) and 8 weeks of aerobic training (36). However, the authors of these studies classified this hypertrophy as physiological due to the short period of administration of L-NAME and the long period of aerobic activity.

The administration of low (7.5 mg·kg^-1^·day^-1^) and moderate (25 mg·kg^-1^·day^-1^) doses of L-NAME for 10 to 24 weeks, whether associated with aerobic exercise or not, was able to promote cardiac remodeling (7,37). These findings suggest that the decrease of nitrite and nitrate (NO metabolites) and guanosine monophosphate cyclic (GMPc) affect the development of cardiac hypertrophy. The most likely factor is the systemic deficiency of NO, which leads to a decrease in the blood supply to the heart, promoting ischemia, the development of local NO-deficient metabolic changes in the cardiomyocytes, and increasing fibrosis factors (7).

In addition to myocyte hypertrophy, another element involved in the cardiac remodeling process is interstitial connective tissue. Increases in collagen content may cause myocardial dysfunction due to impairment of the ventricular compliance, as well as changes in cardiac geometry. The literature suggests that decreased NO synthesis promotes the activation of neurohormonal systems and growth-promoting factors that could induce myocardial fibrosis (38). Cardiac hypertrophy refers to the process of cardiac thickening and remodeling, and may be due to cardiac pathology or long-term exercise training. Our results showed that the association of low-dose L-NAME and chronic aerobic exercise promoted structural alterations that corresponded to physiologic cardiac hypertrophy since we were unable to identify any pathological changes in the myocardial structure (absence of collagen deposition or CSA elevated), suggesting that this association prevented maladaptive myocardial remodeling. Thus, a pressure or volume overload causes initial hypertrophy, which represents a compensatory mechanism for maintaining cardiac function (39), but if these stimuli persist, structural and functional cardiac anomalies could develop. An explanation for our results may be related to aerobic exercise protocol, since chronic aerobic exercise when associated with low-dose L-NAME was only able to promote physiological alterations (no signs of arterial hypertension and cardiac hypertrophy, as well as contractile function preserved) despite a significant cardiac overload evidenced by the elevated LVEDP.

Myocardial function was also evaluated by studying isolated cardiomyocytes. In agreement with our initial hypothesis, ExL improved the cardiomyocyte contractile function characterized by reduced times to 50% shortening and relaxation without alteration in fractional shortening. Considering the intensity used in the present study (80% of speed), we suggest that chronic aerobic exercise, even at higher intensity, may promote positive effects on cardiomyocyte contractile function. In the cardiomyocyte, Ca^2+^ actively participated in several intracellular functions, including excitation-contraction coupling (40). Thus, changes in Ca^2+^ handling were commonly present in the myocardial dysfunction resulting from pathological remodeling. Interestingly, our data also revealed that the association of chronic aerobic exercise with low-dose L-NAME increased the time to 50% Ca^2+^ peak without alterations on Ca^2+^ amplitude and the time to 50% Ca^2+^ decay, despite the improved cardiomyocyte contractility. Ca^2+^ acts as a second messenger that regulates different processes in the cardiac myocytes, mainly contributing to the electrical and contractile activity and the mechanism of contraction-excitation. Carneiro-Junior et al. (22) identified increases in Ca^2+^ transient amplitude and decreases in time to 50% decay in myocyte from hypertensive rats after 8 weeks of aerobic exercise. The same authors reported a decrease in time to 50% Ca^2+^ decay, which probably indicates an enhanced reuptake function of the sarcoplasmic reticulum during diastole (22). The reduction of time to 50% shortening and relaxation without alteration in fractional shortening provisionally promoted a positive effect on contractile function. This improvement was accompanied by an elevated time to 50% Ca^2+^ peak (systolic parameter), suggesting that low-dose L-NAME when associated with chronic aerobic exercise induced an elevation on responsivity to this systolic Ca^2+^.

In summary, the association of chronic aerobic exercise with low doses of L-NAME prevented cardiac pathological remodeling and induced improvement in cardiomyocyte contractile function; however, this association did not alter the myocyte affinity and sensitivity to intracellular Ca^2+^ handling.

### Study limitations

Although it is not an unusual strategy, we understand that one limitation of our study could be regarding the group's selection strategy. Possibly, the ideal strategy would be to include just 'runner rats' equally in all groups. It is not possible to affirm our strategy could have biased the results, even if partially. As the aerobic capacity is essentially caused by cardiovascular improvements, another limitation of our study was not measuring important causal factors of aerobic capacity (e.g., VO_2_max, ventilatory parameters, bradycardia, oxidative metabolic enzymes).
